# Time to address ethnic inclusivity in children & young People's research

**DOI:** 10.1016/j.eclinm.2021.100973

**Published:** 2021-06-18

**Authors:** Babla K, Akindolie O, Gupta A

**Affiliations:** King's College Hospital NHS Foundation Trust, Denmark Hill, London SE9 9RS, UK

Recent discourse has highlighted the burden of health inequalities on ethnic minorities. There is a lack of representation of children and young people (CYP) from ethnic minorities in clinical research, making the significance of trial outcome data for these patients unclear[1].

Race, ethnicity, environmental and genetic factors are determinants of degree of response to drug therapy [2,3]. Specific health beliefs may also affect adherence to therapies, and therefore their pharmacological response. Equitable representation is important to fully understand the effect of these factors on health outcomes.

Within the context of asthma in CYP we discuss problems posed by differential representation in research. We propose some specific areas for improvements in order to redress this imbalance.

Black patients with asthma have higher rates of exacerbation, admission to hospital and death than White patients [3]. Despite this increased health burden and evidence showing racial differences in response to common therapies, ethnic minority patients are underrepresented in research [2,3]. Insufficient data examining the effects of therapies may lead to ‘standardised treatment pathways’ exacerbating inequity by exposing CYP to non-beneficial therapies, or denying them access to beneficial therapies.

The impact of poor representation in early studies is emerging. A recent study found that compared to White patients, Black patients have three times the frequency of hypoxaemia not detected by pulse oximetry [4]. This may have been detected earlier, had initial studies included and reported on ethnic minority data. Furthermore, as ethnicity data is often not collected or reported, it risks undermining its importance.

A recent UK Government report recommended: “we need more Black and Asian people to participate in health trials so that medical research will be based on data that comes from the whole population” [6]. Directly addressing representation at a systemic level has great potential for improving health inequalities. We propose measures that may ameliorate this problem in research involving CYP, though our recommendations are applicable to the wider research setting.

We agree with previous work suggesting a multi-faceted approach to increasing representation of ethnic minority groups in research, encompassing recommendations on reporting standards, funding interventions to clarify the reasons for under-representation, encouraging policies where study participants should represent the ethnic composition of the local community, and encouraging representation of ethnic minorities in leadership roles [1].

Underrepresentation of ethnic minorities in clinical research and journal editorial teams is widely reported [5]. Representation in senior leadership roles such as research directors, senior executive, chief investigators, principal investigators, NIHR Fellowship, Professorship or roles within research organisations enables younger researchers to feel empowered, whilst those in role are able to develop valuable skills and support researchers from ethnic minorities.

Making ethnic inclusivity the norm in health research requires a shift in culture and attitudes. Adequate representation in research teams will build trust, confidence, and improve understanding of the barriers to participation for CYP and families from ethnic minorities. This will help develop questions and research methods that elicit minority groups’ needs and experiences. Researchers from ethnic minorities may have unique roles in recruiting participants, collecting data and disseminating findings, whilst increasing community awareness and engagement with research.

We suggest introducing specific questions in funding applications regarding the inclusion of ethnic minority groups, in order to hold researchers accountable. These could ask about additional steps researchers will take to recruit CYP and families from ethnic minorities. For example, whether they will ensure use of translation services, preparing patient information and consent documents in different languages. These questions will require researchers to demonstrate they have clarified which ethnic groups they may be recruiting from, and therefore which approaches and resources are required. We recommend improved reporting of the ethnicities of CYP recruited to studies in order to understand what outcomes mean for patients from ethnic minorities. Stakeholders must commit to strengthening ethnic minorities’ engagement and actively taking steps to address existing issues.

Finally, we suggest increased involvement of CYP and families from ethnic minorities in research design, and institutional support to help researchers engage with research participants meaningfully. Patient champions/groups may suggest approaches that help researchers engage with ethnic minorities by understanding their cultural and health beliefs, language and communication needs, or being able to engage remotely. Increasing representation of ethnic minority groups may help drive this, but requires pre-defined strategies on evaluating the impact of CYP involvement. This may require creative approaches to engage with CYP from ethnic minorities who may otherwise be unable or reluctant to participate.

Differential health outcomes are noted for CYP from ethnic minorities, in the context of under-representation in research and a lack of clarity in reporting. We highlight the need for increased involvement of CYP and families from ethnic minorities in research funding, policy and design, as well as an increased drive to recruit from ethnic minorities and clearly report data. These steps may contribute to better understanding of the effects of health interventions in CYP from ethnic minorities, and potentially improve differential outcomes. There is a compelling clinical, moral and ethical case for prioritising and definitively addressing this issue.

## Declaration of Competing Interest

The authors have no declarations of interests to make.

## References

[1] Osuafor CN, Golubic R, Ray S. (2021). Ethnic inclusivity and preventative health research in addressing health inequalities and developing evidence base. EClinicalMedicine [Internet].

[2] Koo S, Gupta A, Fainardi V, Bossley C, Bush A, Saglani S (2016). Ethnic variation in response to im triamcinolone in children with severe therapy-resistant asthma. Chest [Internet].

[3] Nyenhuis SM, Krishnan JA, Berry A, Calhoun WJ, Chinchilli VM, Engle L (2017). Race is associated with differences in airway inflammation in patients with asthma. J Allergy Clin Immunol [Internet].

[4] Sjoding MW, Dickson RP, Iwashyna TJ, Gay SE, Valley TS. (2020). Racial bias in pulse oximetry measurement. N Engl J Med.

[5] Smart A, Harrison E. (2017). The under-representation of minority ethnic groups in UK medical research. Ethn Heal.

[6] UK Government. Commission on race and ethnic disparities: the report [Internet]. London; 2021. Available from: https://www.gov.uk/government/news/the-commission-on-race-and-ethnic-disparities-statement.

